# Positive Expression of Retinol-Binding Protein 4 Is Related to the Malignant Clinical Features Leading to Poor Prognosis of Glioblastoma

**DOI:** 10.1155/2022/5435523

**Published:** 2022-12-26

**Authors:** Xinqing Deng, Jian Ren, Zhongsheng Bi, Zhenghao Fu

**Affiliations:** ^1^Guangdong 999 Brain Hospital, Guangzhou 510000, China; ^2^Department of Neurological Diseases Center, The Third Affiliated Hospital of Chongqing Medical University (Gener Hospital), Chongqing 401120, China

## Abstract

**Backgrounds:**

Retinol-binding protein 4 (RBP4) is a monomeric-binding protein belonging to the lipocalin protein family, which has been reported to be dysregulated in several malignancies such as breast cancer and lung cancer. However, the expression and function of RBP4 in glioblastoma (GBM) are completely unknown.

**Materials and Methods:**

TCGA datasets were used for analyzing the mRNA level of RBP4 in GBM and its clinical relevance. A retrospective GBM cohort (*n* = 73) was enrolled from our hospital to test the protein expression profile of RBP4 in GBM tissues as well as its correlation with patients' prognoses. Two human GBM cell lines, LN229 and U251, were collected to conduct overexpression and knockdown experiments targeting RBP4. The tumor-related effects of RBP4 in GBM were finally evaluated by proliferation and invasion assays.

**Results:**

Both the higher mRNA level and protein level of RBP4 in GBM tissues were significantly correlated with poorer patients' overall survival. Multivariate analysis identified RBP4 as a novel independent prognostic predictor in GBM patients. Overexpression of RBP4 resulted in enhanced GBM proliferation capacity, which was consistent with clinical findings on the positive correlation between RBP4 level and tumor size. Meanwhile, overexpressing RBP4 promoted GBM cell migration and invasion, while silencing RBP4 led to the opposite results.

**Conclusions:**

RBP4 overexpression in tumor tissues is correlated with poorer prognosis of GBM patients, which functions by promoting GBM proliferation and invasion, thus, may serve as an invaluable predictive biomarker and therapeutic target.

## 1. Introduction

The most common malignancy of the central nervous system in adults is glioma, which originates in the brain or glial tissues. Pathologically, gliomas can be further classified as astrocytomas, ependymomas, oligodendrogliomas, and glioblastomas (GBM), and so on. Among them, GBM is the most aggressive type of glioma [1]. GBM treatment depends on size, location, patient status, and other clinical and pathological factors. Major therapies include surgery, radiotherapy, and chemotherapy [2]. Due to the aggressive phenotype of GBM, the median survival time of patients is only 15 months despite advances in therapeutic development [3]. Failing to precisely predict patients' survival is another contributor for unsatisfied outcomes. Thus, there is still a great need to further understand the pathology of GBM and to employ more effective methods for prognostic prediction as well as disease treatment.

Retinol-binding protein 4 (*RBP4*) is a monomeric-binding protein of 21 kilo Dalton, which belongs to the lipocalin family and was initially identified as the specific carrier for retinol in plasma [4]. Dysregulated RBP4 results in numerous diseases such as retinal dystrophy, iris Coloboma, comedogenic acne syndrome, and microphthalmia [5, 6]. Moreover, an aberrant level of plasma RBP4 has been discovered in several malignancies. For example, a significant elevation in the serum level of RBP4 was found in pancreatic cancer patients, which was significantly decreased after operation than before operation [7]. Of note, the serum level of RBP4 showed no statistically different between patients with pancreatic adenocarcinoma or chronic pancreatitis, indicating it may not be a suitable biomarker for pancreatic cancer [8].

However, the fact that RBP4 is altered in several tumor types is contradictory. On the other hand, the serum concentration of RBP4 in ovarian cancer patients was significantly lower than that in healthy volunteers, according to mass spectrometry data [9]. On the other hand, Cheng et al. reported a significantly higher serum RBP4 level in ovarian cancer patients than those in healthy individuals [10]. Similar contradictory findings were reported in renal cell carcinoma (RCC). Choi et al. reported that RBP4 was higher in RCC cases than that in healthy controls [11], while the lower serum RBP4 level was correlated with unfavorable overall survival (OS) and disease-free survival (DFS) according to Sobotka's data [12].

Moreover, it appears that the expression pattern of RBP4 in solid tumor tissues differs from its serum concentrations. For example, patients with breast cancer may showed higher serum RBP4 level [13], although another study found no statistically significant association between serum RBP4 and breast cancer development [14]. Intriguingly, bioinformatic analyses demonstrated a lower expression of RBP4-mRNA in breast cancer tissues, and lower RBP4 indicated poorer overall survival of breast cancer patients [15]. Similarly, hepatocellular carcinoma (HCC) patients with higher serum RBP4 levels exhibited poorer OS and DFS compared to those with lower serum RBP4 levels [16]. In contrast to the serum level, RBP4 expression was low in HCC tissues compared with normal tissues, and low RBP4 expression levels were associated with advanced tumor stages and poorer overall survival [17].

Therefore, RBP4 may play multiple roles in different types of cancer. Here, we aimed to initially investigate the expression and potential function of RBP4 in GBM from both the TCGA dataset and our retrospective cohort. A univariate and multivariate analysis was conducted to evaluate the clinical significance and prognostic predictive role of RBP4 in GBM, which revealed its independent prognostic effect. Additionally, we assessed the tumor-related function of RBP4 in GBM cell lines by testing their proliferation, migration, and invasion after RBP4 interference.

## 2. Materials and Methods

### 2.1. Online Data Mining

The RBP4-mRNA in GBM tissues was extracted from the TCGA dataset and presented as FPKM (fragments per kilobase of transcript per Million) according to the RNA-seq data. The Kaplan–Meier method was used to evaluate the prognostic effect of RBP4-mRNA. MethSurv web tool (version MethSurv©2017; https://biit.cs.ut.ee/methsurv/) was used to perform survival analysis based on RBP4 methylation using TCGA data [18].

### 2.2. Patients' Information and Ethics

We retrospectively enrolled a cohort of GBM cases (*n* = 73) from our hospital, including 31 females and 42 males. All cases were pathologically diagnosed as single lesion GBM without other malignancy history. All cases underwent surgical resection (39 cases underwent local resection, 16 cases with radical resection, and 18 cases with lobectomy). None of the cases possessed distant metastasis before surgery. None of the cases underwent neoadjuvant chemotherapy or radiotherapy, but 12 cases underwent postoperative adjuvant chemotherapy while the other 61 cases rejected chemotherapy or unknown. The median age was 63 years old at the time of diagnoses. The median tumor size was 4.3 cm according to pathological records. As for the tumor location, 13 tumors located in the parietal lobe, 25 tumors in temporal lobe, 32 cases in the frontal lobe, and 3 unclear based on the surgical record. This study was approved by the Ethic Committee of Guangdong 999 Brain Hospital. Written informed consent was provided by each patient or immediate family member at the start of the study, and all procedures in this study were performed in accordance with the Declaration of Helsinki.

### 2.3. Immunohistochemistry (IHC) Staining

A total of 73 GBM tissue samples were obtained, and tissue sections were treated for antigen retrieval using EDTA buffer according to a standard procedure [19], and then the tissues were incubated with a primary anti-RBP4 antibody (1 : 300, sc-48384, Santa Cruz Biotechnology) at 4°C overnight. Next, the sections were incubated with a secondary antibody and then visualized using diaminobenzidine (DAB) and hematoxylin counterstaining. The immunohistochemical results were evaluated by two pathologists and defined as negative staining unless there were more than 50% positively stained cells with moderate to dark staining intensity. The two pathologists who analyzed IHC data were blinded to the patient's information in this study.

### 2.4. Cell Lines and Transfection

Human originating GBM cell lines LN229 and U251 were obtained from ATCC (American Type Culture Collection) and cultured in DMEM medium (Invitrogen) supplemented with 10% FBS at 37°C in a humidified atmosphere with 5% CO2. Knockdown and overexpression were achieved by using Lipo3000 transfection reagent (Thermo Fisher Scientific) according to the manufacturer's instructions. The siRNA targeting human RBP4 was synthesized by GenePharma (Shanghai, China) as described by others [20]. The cDNA of RBP4 was cloned into a pcDNA3.0 vector by GenePharma. For the control group, cells were treated with transfection reagent and underwent the same experimental procedure.

### 2.5. Western Blot (WB)

Total protein in transfected cells was extracted with RIPA buffer (Beyotime, Shanghai, China), and its concentration was tested via a BCA protein assay kit (Thermo Fisher Scientific). Protein samples were then separated by SDS-PAGE and transferred to PVDF membrane (Millipore, MA, USA). Afterwards, the membrane was blocked with 5% nonfat milk and incubated with primary antibodies (RBP4, 1 : 1000, sc-48384;beta-actin, 1 : 2000, sc-8432; both from Santa Cruz Biotechnology) at 4°C overnight followed by secondary antibody incubation for 1 h in room temperature. The immunoreactivity was visualized by ECL reagents [21].

### 2.6. MTT (3-(4,5-Dimethylthiazol-2-yl)-2,5-Diphenyltetrazolium Bromide) Assay

The MTT assay was used to measure the proliferation of transfected cells. Briefly, 200 *μ*L of transfected cells were incubated in a 96-well plate (2,000 cells/well) for 1–5 days. On each day, 20 *μ*L of MTT solution (5 mg/mL) was added to each well and incubated for 4 h. The optical density was measured at 570 nm using a microplate reader. Each experiment was conducted for three independent times.

### 2.7. Colony Formation

The colony formation assay was used to evaluate the clone-forming capacity of transfected cells. Briefly, 500 cells were inoculated into a 6-well plate and cultured for 14 days, during which period the medium was changed every five days. Afterwards, the colonies were immobilized with methanol and dyed with 0.2% crystal violet. Finally, the clone number was counted. Each experiment was conducted for three independent times.

### 2.8. Transwell Assay

Transfected cells were resuspended in serum-free DMEM medium (100 *μ*L, containing 5,000 cells) and seeded into the upper chamber of the 24-well plate Transwell chamber (Corning, Cambridge, MA, USA), while the lower was added with 200 *μ*l DMEM containing 10% FBS. Cells were cultured in 5% CO_2_ at 37°C for 48 h, and then, the chamber was taken out, cells on the upper surfaces of the chamber were wiped off with cotton swabs, and cells from the bottom surfaces were fixed and stained with crystal violet for 15 min. Migrated cells were counted under an inverted microscope. The cell invasion assay was also conducted as described above except that the membrane was precoated with diluted Matrigel (BD Biosciences, San Jose, CA, USA). In addition, the cell seeding number for invasion experiments was 20,000 cells/well. Each experiment was conducted for three independent times.

### 2.9. Statistics

Statistical analyses were carried out using SPSS 16.0 software. The cancer-specific survival was defined as the length of time from the date of diagnosis to the date of death from GBM. The overall survival was defined as the length of time from the date of diagnosis to the date of death from any causes. We also added the descriptions in our revised main text. Kaplan–Meier method was used to generate survival curves and compared by the log-rank test. Multivariable Cox regression analysis was used to test the independent prognostic effects of variables. Two tails of the student's *t*-test was used to compare the differences between groups in cellular experiments. *P* < 0.05 was chose to be statistically significant.

## 3. Results

### 3.1. High RBP4-mRNA Level in GBM Tissues Indicates Poor Prognosis

**Figure 1:**
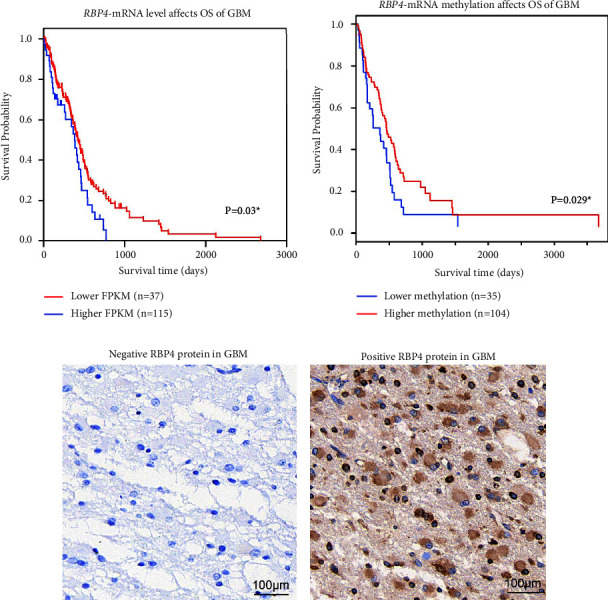
Transcription, methylation, and protein expression of RBP4 in GBM. (a) The mRNA level of RBP4 in GBM tissues was retrieved from TCGA datasets and divided into low level or high level according to the FPKM (fragments per kilobase of transcript per million). Then overall survival (OS) curves were plotted using Kaplan–Meier method and compared by the log-rank test, which revealed that higher mRNA level of RBP4 was correlated with poorer OS (*P*=0.03). (b) The methylation of RBP4 in GBM tissues was obtained from MethSurv web tool (https://biit.cs.ut.ee/methsurv/) and subgrouped as lower methylation level and higher methylation level. Survival curves showed that patients with lower RBP4 methylation level had poorer prognosis (*P*=0.029). (c) Representative negative protein expression of RBP4 in GBM tissues. Magnification: 400X. (d) Representative positive protein expression of RBP4 in GBM tissues. Magnification: 400X.

We first evaluated the potential clinical relevance of RBP4 by retrieving its mRNA level from TCGA datasets. Accordingly, GBM patients with low RBP4-mRNA showed poorer overall survival (Figure 1(a), *P*=0.03). Meanwhile, higher methylation of RBP4-mRNA was also correlated with poorer overall survival (Figure 1(b), *P*=0.029). Considering lower mRNA levels were generally correlated with higher methylation, the mRNA data and methylation data were consistent.

### 3.2. Protein Expression of RBP4 in GBM Tissues

To further explore the role of RBP4 in GBM, we next tested its protein expression pattern in GBM tissues from a retrospective cohort (*n* = 73). As revealed by IHC results, GBM tissues exhibited distinct RBP4 protein expression levels. According to the immunoreactivity, 31 cases were classified as negative RBP4 expression (Figure 1(c)), while the other 42 cases were defined as positive RBP4 protein expression (Figure 1(d)). Interestingly, gliomas with larger tumor sizes were more prevalent to exhibit positive RBP4 expression compared with the smaller ones (*P* < 0.001, Table 1), indicating that RBP4 may participate in GBM progression.

### 3.3. Prognostic Factors of GBM Cohort

**Figure 2:**
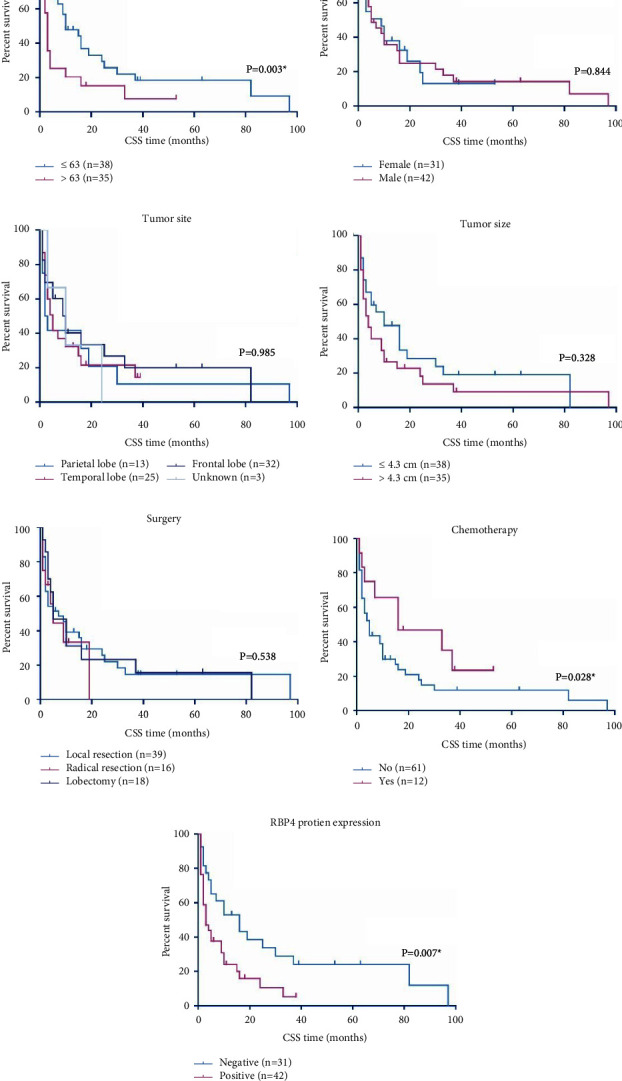
cancer-specific survival curves of enrolled GBM cohort. The cancer-specific survival (CSS) of GBM patients were analyzed according to patients' age (a), sex (b), tumor location (c), tumor size (d), surgery pattern (e), adjuvant chemotherapy (f), and RBP4 protein expression in tumor tissues (g).

Kaplan–Meier survival curves were next plotted to assess prognostic factors of GBM (Figure 2). The median age was 63 years old (ranging from 34–79 years old) at the time of diagnosis. The median follow up time was 3 months, ranging from 0–97 months. According to the log-rank test (Table 2) elder patients (>63 years old) showed poorer prognoses than younger patients (≤63 years old). The median cancer-specific survival (CSS) time of younger patients was 23.6 ± 5.6 months, while decreased to 7.2 ± 2.6 months of elder patients (*P*=0.003). Consistently, the median overall survival (OS) time of younger patients was 23.6 ± 5.6 months, while it decreased to 5.8 ± 2.1 months of elder patients (*P* < 0.001). Although patients underwent chemotherapy showed better CSS (24.8 ± 5.9 months) than those without chemotherapy (13.8 ± 3.7 months, *P*=0.028), the OS time showed no statistically significant difference (22.3 ± 6.1 months vs 13.1 ± 3.5 months, *P*=0.071). The difference between OS and CSS can be partially explained by the fact that although chemotherapy can suppress tumor recurrence, it may have systematically adverse effects and may be harmful to the human body system. Importantly, the median CSS time of patients with negative RBP4 expression was 26.6 ± 6.8 months, while it decreased to 7.3 ± 1.7 months of those with positive RBP4 expression (*P*=0.007). As for the OS, the median OS time of patients with negative RBP4 expression was 22.6 ± 5.9 months, while it decreased to 6.9 ± 1.6 months of those with positive RBP4 expression (*P*=0.013). According to our cohort, patients' sex, tumor location, tumor size, and surgery pattern had no statistically significant effect on the prognosis of GBM (all *P* > 0.05).

Since univariate data indicated that RBP4 has significant effect on the prognosis of GBM patients, its prognostic value was further validated by multivariate analysis (Table 3). As a result, comparing with negative RBP4 expression, positive RBP4 exerts a hazard ratio as 1.832 (95% CI 1.040–3.226, *P*=0.036), highlighting its role as a novel independent prognostic predictive biomarker. Meanwhile, patients' age (HR = 2.346, 95% CI 1.321–4.167, *P*=0.004) and chemotherapy (HR = 0.325, 95% CI 0.146–0.723, *P*=0.006) were also identified as independent factors affecting the CSS of GBM patients. Similarly, we conducted multivariate analysis targeting patients' overall survival (Table 4). As a result, both elder age (HR = 2.16, 95% CI 1.27–3.68, *P*=0.004) and a higher RBP4 (HR = 1.72, 95% CI 1.11–2.65, *P*=0.025) can independently help predict a worse overall survival of GBM.

### 3.4. RBP4 Can Promote Proliferation, Migration, and Invasion of GBM Cells

**Figure 3:**
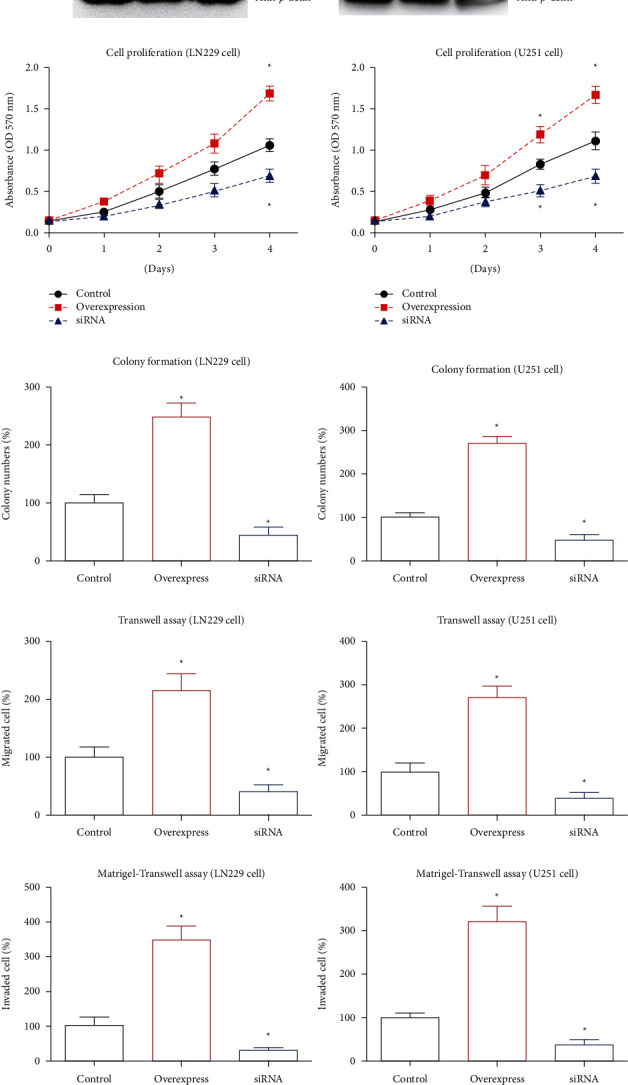
RBP4 promotes GBM proliferation, migration, and invasion. (a) LN229 and U87 cells were transfected with pcDNA3.0-RBP4 plasmids (overexpress), transfection reagent (control), or RBP4-siRNA, respectively. The transfection efficiencies were tested by western blot. (b) Proliferation of transfected LN229 and U87 cells were tested via MTT method. The proliferation curves indicated that overexpressing RBP4 promotes cell proliferation while silencing RBP4 inhibits cell proliferation. (c) Colony formation assays were also conducted to evaluate cell proliferation capacity. Comparing with control cells, clone-forming capacities of RBP4-overexpressed cells were significantly enhanced while impaired in RBP4-knockdown cells. (d) Migration capacities of transfected cells were tested by Transwell assay, which demonstrated that RBP4 can positively regulation the migration process. (e) Invasion abilities of GBM cells were assessed by Matrigel–Transwell method. Accordingly, overexpressing RBP4 increased the number of invaded cells while silencing RBP4 decreased the number of invaded cells. Data were presented as mean ± SD. Each experiment was conducted for three independent times. ^*∗*^*P* < 0.05 compared to control group.

Here, we also conducted cellular experiments to validate the tumor-related role of RBP4 in GBM. The transfection efficiencies of overexpression and knockdown were tested by Western blot (Figure 3(a)). Comparing with control cells, RBP4-overexpression cells showed 3.8 folds and 4.1 folds increase on the RBP4 protein levels in LN229 cells and U251 cells, respectively. In contrast, siRNA transfection led to an 83% and 69% decrease on the RBP4 expression levels compared with the control groups in LN229 and U251 cells, respectively. After then, the cell viability was analyzed by the MTT assay and colony formation assay (Figures 3(b) and 3(c)). Accordingly, overexpressing RBP4 can significantly enhance both LN229 and U251 cell proliferation. Oppositely, silencing RBP4 resulted in impaired cell viability. Furthermore, we assessed the effects of RBP4 on GBM migration and invasion via Transwell strategies (Figures 3(d) and 3(e)). As a result, sRBP4-overexpression enhanced cell migration and invasion, while RBP4-knockdown inhibited the migration and invasion processes of both GBM cell lines.

## 4. Discussion

Since RBP4 was originally identified as a circulation protein in plasma and showed a predictive role of in several malignancies including lung cancer [22] and colon cancer [23], we were interested to investigate its role in GBM. However, our preliminary ELISA data showed no statistically significant difference regarding the serum level of RBP4 in GBM patients and healthy volunteers (data not shown). Interestingly, a higher RBP4 level in stool was also identified as a biomarker for noninvasive detection of colorectal adenomas with a high risk of progression [24]. Since literature research demonstrated that RBP4 can also be localized in solid organs such as livers, and its abnormal expression in tissues was also correlated with malignancies. For example, immunohistochemistry results demonstrated that RBP4-protein expression levels in ovarian cancer tissues were higher than those in normal ovarian tissue and exerted prognostic predictive roles [10, 25]. Consistently, our data demonstrated that higher RBP4- mRNA and protein expression in GBM tissues was correlated with poorer survival after surgical treatment.

Besides the expression levels, online data mining revealed that the methylation of RBP4 in tumor tissues was positively correlated the overall survival of GBM patients (*P*=0.025). Similarly, methylation of RBP4 in esophageal squamous cell carcinoma seems more frequent than that in adjacent esophagus tissues, however, the difference was not statistically significant (*P*=0.08) [26]. Nevertheless, dysregulated methylation may help explain the abnormal expression of RBP4 in malignancies, which deserve more investigation.

A recent study reported that downregulating RBP4 in colon cancer cells decreased the fraction of cancer stem cells [27], therefore we also tested the cellular effects of RBP4 in GBM cells. According to the MTT assay and colony formation assay, knockdown of RBP4 resulted in impaired proliferation capacity of GBM cells, while RBP4-overexpression showed the opposite effects. Our data was consistent with the reported functions of RBP4 in prostate cancer cells [20] and ovarian cancer cells [25]. Besides proliferation, RBP4 also participates in the metastasis of malignancies. For example, genomic studies revealed that liver metastases from the colon adenocarcinomas showed significantly higher RBP4 transcript than that in paired primary colorectal carcinomas [28]. According to the data by Uehara et al. tissue expression level of RBP4 was identified to be increased in a prostate cancer bone metastasis mice model [20]. By comparing different cell lines, Wang et al. also demonstrated that RBP4 protein was overexpressed in HCC cell lines compared with normal liver cell line and correlated with metastatic potential [16]. Additionally, RBP4 was reported to enhance the metastatic potential of breast cancer through both direct effect on cancer cells and through impairing endothelial blood vessels within the tumor [29]. According to the migration and invasion tests, our data also revealed a similar conclusion on the potential metastasis-promoting effect of RBP4 in GBM, highlighting that RBP4 may become new therapeutic targets for both tumor growth and metastasis. Indeed, serum level of RBP4 was upregulated after LA-12, a platinum-based anticancer agent, treatment in rat models [30]. Similarly, Phase I clinical trials of LA-12 indicated that circulating RBP4 levels correlated well with platinum levels in the human plasma of 12 randomly selected patients with solid tumors [30]. Since retinol metabolism is closely associated with many malignancies, targeting RBP4 may represent a novel direction for drug development.

Our study has several limitations. First, RBP4 was closely correlated with metabolism [27] and has been recognized as a link between adiposity and cancer [31], however, here we didn't find any significant correlation between the body mass index with the RBP4 levels. Whether RBP4 participate in high-fatdiet-induced malignancies remains further investigation. Second, our study lacks sufficient clinical cohorts to validate the conclusions generated by the retrospective cohort with limited case numbers. Third, we did not fully dig into RBP4's mechanism for affecting GBM progression through systematic molecular biology experiments.

## 5. Conclusions

In conclusion, our results suggested that RBP4-overexpression in tumor tissues is correlated with poorer prognosis of GBM patients. Therefore, RBP4 may serve as an invaluable predictive biomarker and therapeutic target for malignancies.

## Figures and Tables

**Table 1:** The correlations between clinical parameters and RBP4 expression in GBM.

Variables	Patients	RBP4 expression		*P* values
	(*n* = 73)	Negative (*n* = 31)	Positive (*n* = 42)	
*Age (years)*				
≤63	38	20	18	0.067
>63	35	11	24	
*Sex*				
Female	31	13	18	0.937
Male	42	18	24	
*Location*				
Parietal lobe	13	5	8	0.909
Temporal lobe	25	12	13	
Frontal lobe	32	13	19	
Unknown	3	1	2	
*Tumor size*				
≤4.3 cm	38	27	14	<0.001^*∗*^
>4.3 cm	35	7	28	
*Surgery*				
Local resection	39	15	24	0.037^*∗*^
Radical resection	16	4	12	
Lobectomy	18	12	6	
*Chemotherapy*				
No	61	25	36	0.564
Yes	12	6	6	

*Note. * ^*∗*^Statistically significant by the chi-square test or Fisher's exact test.

**Table 2:** Univariate analyses of cancer-specific survival and overall survival of GBM patients.

Variables	Patients	Cancer-specific survival		Overall survival	
	(*n* = 73)	Months	*P* value	Months	*P* values
*Age (years)*					
≤63	38	23.6 ± 5.6	0.003^*∗*^	23.6 ± 5.6	<0.001^*∗*^
>63	35	7.2 ± 2.6		5.8 ± 2.1	
Sex					
Female	31	11.8 ± 3.2	0.844	11.2 ± 3.1	0.747
Male	42	17.5 ± 4.8		16.2 ± 4.5	
*Location*					
Parietal lobe	13	16.5 ± 8.6	0.985	13.6 ± 7.4	0.971
Temporal lobe	25	11.4 ± 2.9		10.8 ± 2.8	
Frontal lobe	32	17.4 ± 5.5		15.1 ± 4.7	
Unknown	3	12.3 ± 6.2		12.3 ± 6.1	
*Tumor size*					
≤4.3 cm	38	19.5 ± 5.2	0.328	15.8 ± 4.3	0.846
>4.3 cm	35	13.8 ± 4.7		13.8 ± 4.7	
*Surgery*					
Local resection	39	19.8 ± 5.4	0.538	16.9 ± 4.7	0.262
Radical resection	16	6.6 ± 2.1		5.3 ± 1.8	
Lobectomy	18	15.7 ± 6.6		14.6 ± 6.2	
*Chemotherapy*					
No	61	13.8 ± 3.7	0.028^*∗*^	13.1 ± 3.5	0.071
Yes	12	24.8 ± 5.9		22.3 ± 6.1	
*RBP4 expression*					
Negative	31	26.6 ± 6.8	0.007^*∗*^	22.6 ± 5.9	0.013^*∗*^
Positive	42	7.3 ± 1.7		6.9 ± 1.6	

*Note. * ^*∗*^Statistically significant.

**Table 3:** Multivariate analysis of the independent CSS prognostic factors of GBM patients.

Variables	HR	95% CI	*P* values
Age	2.346	1.321–4.167	0.004^*∗*^
Chemotherapy	0.325	0.146–0.723	0.006^*∗*^
RBP4 expression	1.832	1.040–3.226	0.036^*∗*^

*Note. * ^*∗*^Statistically significant.

**Table 4:** Multivariate analysis of the independent OS prognostic factors of GBM patients.

Variables	HR	95% CI	*P* values
Age	2.164	1.274–3.676	0.004^*∗*^
RBP4 expression	1.716	1.108–2.649	0.025^*∗*^

*Note. * ^*∗*^Statistically significant.

## Data Availability

The data are made available from the corresponding author upon reasonable request.
